# Behavioral and Psychological Symptoms of Dementia in Persons with Autism Spectrum Disorder”

**DOI:** 10.1002/alz70858_097017

**Published:** 2025-12-24

**Authors:** Kok Wai Tay, Joanne Rou Heng Chin, Shu Ping Lim, Zi Ching Tan, Ponnusamy Subramaniam

**Affiliations:** ^1^ Tunku Abdul Rahman University, Kampar, Malaysia; ^2^ National University of Malaysia, Kuala Lumpur, Malaysia; ^3^ Clinical Psychology Programme and Centre for Healthy Aging and Wellness, Faculty of Health Sciences, Universiti Kebangsaan Malaysia, Kuala Lumpur, Kuala Lumpur, Malaysia

## Abstract

**Background:**

The prevalence of dementia is increasing globally, significantly impacting caregivers due to the behavioral and psychological symptoms of dementia (BPSD). One of the factors that compound this issue is the lack of reliable diagnostic tools in the past, leading to many undiagnosed neurodevelopmental disorders among the aging population. The overlapping symptoms between autism spectrum disorder (ASD) and dementia often make it challenging to identify undiagnosed ASD in people with dementia (PWD), potentially leading to the misattribution of certain BPSD to dementia alone when they may partly stem from underlying ASD. Emerging evidence highlights a concerning intersection between neurodevelopmental disorders and dementia. For example, a study reported 18.5% of PWD in memory clinics exhibit autism symptoms, and individuals with autism under 65‐year‐old are 2.6 times more likely to be diagnosed with dementia than the general population. Early‐onset dementia also follows the same trend, with a 4.04% occurrence in autistic population compared to 0.97% in the healthy population.

**Method:**

This review examines current literature. Data will be extracted and analysed through thematic analysis to identify key themes and gaps.

**Result:**

Historical links between autism and dementia further imply these associations; childhood disintegrative disorder, was once labeled as dementia infantilis. Preliminary studies have revealed significant associations between autism spectrum symptoms and both the severity of BPSD and dementia. Another study also found that severe dementia correlates with increased neurodevelopmental symptoms. These findings suggest that understanding BPSD through the lens of autism may offer new insights into its underlying mechanisms, providing alternative explanations for the behavioral patterns observed among people with dementia.

**Conclusion:**

This paper hypothesizes that many PWD may have lived with undiagnosed mild autism, coping adequately until neurodegeneration undermines their learned strategies. Managing mild dementia may also involve addressing comorbid autism symptoms, such as RRB and SCI difficulties. Viewing BPSD partly from the perspective of ASD offers a novel approach to understanding and managing these symptoms. This intersection presents a critical area for future research as current dementia assessments often fail to capture the full spectrum of ASD‐like behaviors, necessitating adaptations in dementia care to address potential comorbidities.

